# Improved methods and resources for paramecium genomics: transcription units, gene annotation and gene expression

**DOI:** 10.1186/s12864-017-3887-z

**Published:** 2017-06-26

**Authors:** Olivier Arnaiz, Erwin Van Dijk, Mireille Bétermier, Maoussi Lhuillier-Akakpo, Augustin de Vanssay, Sandra Duharcourt, Erika Sallet, Jérôme Gouzy, Linda Sperling

**Affiliations:** 1 Institute for Integrative Biology of the Cell (I2BC), CNRS, CEA, Univ. Paris-Sud, Université Paris-Saclay, 91198 Gif-sur-Yvette CEDEX, France; 2 Institut Jacques Monod, CNRS, UMR 7592, Université Paris Diderot, Sorbonne Paris Cité, F-75205 Paris, France; 3 LIPM, Université de Toulouse, INRA, CNRS, Castanet-Tolosan, France; 4 Current address: IRCM, CEA, INSERM UMR 967, Université Paris Diderot, Université Paris-Saclay, 92265 Fontenay-aux-Roses CEDEX, France

**Keywords:** Ciliate, Cap-Seq, TSS, RNA-Seq, Gene annotation, Autogamy, Differential gene expression

## Abstract

**Background:**

The 15 sibling species of the *Paramecium aurelia* cryptic species complex emerged after a whole genome duplication that occurred tens of millions of years ago. Given extensive knowledge of the genetics and epigenetics of *Paramecium* acquired over the last century, this species complex offers a uniquely powerful system to investigate the consequences of whole genome duplication in a unicellular eukaryote as well as the genetic and epigenetic mechanisms that drive speciation. High quality *Paramecium* gene models are important for research using this system. The major aim of the work reported here was to build an improved gene annotation pipeline for the *Paramecium* lineage.

**Results:**

We generated oriented RNA-Seq transcriptome data across the sexual process of autogamy for the model species *Paramecium tetraurelia*. We determined, for the first time in a ciliate, candidate *P. tetraurelia* transcription start sites using an adapted Cap-Seq protocol. We developed TrUC, multi-threaded Perl software that in conjunction with TopHat mapping of RNA-Seq data to a reference genome, predicts transcription units for the annotation pipeline. We used EuGene software to combine annotation evidence. The high quality gene structural annotations obtained for *P. tetraurelia* were used as evidence to improve published annotations for 3 other *Paramecium* species. The RNA-Seq data were also used for differential gene expression analysis, providing a gene expression atlas that is more sensitive than the previously established microarray resource.

**Conclusions:**

We have developed a gene annotation pipeline tailored for the compact genomes and tiny introns of *Paramecium* species. A novel component of this pipeline, TrUC, predicts transcription units using Cap-Seq and oriented RNA-Seq data. TrUC could prove useful beyond *Paramecium,* especially in the case of high gene density. Accurate predictions of 3′ and 5′ UTR will be particularly valuable for studies of gene expression (e.g. nucleosome positioning, identification of cis regulatory motifs). The *P. tetraurelia* improved transcriptome resource, gene annotations for *P. tetraurelia*, *P. biaurelia, P. sexaurelia* and *P. caudatum*, and *Paramecium*-trained EuGene configuration are available through ParameciumDB (http://paramecium.i2bc.paris-saclay.fr). TrUC software is freely distributed under a GNU GPL v3 licence (https://github.com/oarnaiz/TrUC).

**Electronic supplementary material:**

The online version of this article (doi:10.1186/s12864-017-3887-z) contains supplementary material, which is available to authorized users.

## Background

Ciliates are unique among unicellular eukaryotes in making a germ/soma distinction. The germline and somatic functions of chromosomes are respectively ensured by a germline micronucleus (MIC) which undergoes meiosis and fertilization and a somatic macronucleus (MAC) that contains a version of the germline genome stripped of parasitic sequences and optimized for gene expression. The MAC is lost at each sexual cycle and a new one differentiates from a copy of the zygotic nucleus, by reproducible DNA elimination under the control of meiosis-specific RNA interference pathways [1].

Genetics of the ciliate *Paramecium* was pioneered nearly a century ago [2] and this complex unicellular eukaryote has since served as model for a variety of biological processes commonly found in animals, from excitable membranes and swimming behavior [3] to programmed DNA elimination and its epigenetic control during somatic differentiation [4]. The early genetics studies led to the realization that *Paramecium aurelia* is a complex of morphologically identical but reproductively isolated sibling species, renamed *primaurelia, biaurelia, triaurelia, tetraurelia,* etc. [5]. *Paramecium tetraurelia* became the most widely used species for genetics and physiology, because of its convenient growth properties.

*P. tetraurelia* somatic genome sequencing revealed that the present diploid genome was shaped by a series of at least 3 whole genome duplications (WGDs), each WGD being slowly resolved by gene loss over evolutionary time [6]. It was suggested that the *P. aurelia* species complex emerged concomitantly with the most recent WGD [6], a hypothesis confirmed by sequencing two other *aurelia* genomes and *P. caudatum* as outgroup [7, 8]. Custom microarrays were designed to obtain gene expression data for the nearly 40, 000 *P. tetraurelia* protein-coding genes [9]. The data were used to show that gene expression level is a major determinant of gene dosage and protein evolution [10].

The *Paramecium aurelia* species complex is now recognized as an outstanding system to study the consequences of WGD in a unicellular eukaryote [11] and should also prove powerful for investigation of genetic and epigenetic mechanisms that drive speciation. In this context, the MAC genomes of many species are being sequenced. It thus became necessary to develop a pipeline optimized for the *Paramecium* lineage, able to make accurate gene predictions for AT-rich, compact (>80% coding) eukaryotic genomes with unusually small introns (20–30 nt). To this purpose, we generated oriented *P. tetraurelia* RNA-Seq and Cap-Seq data, as input for software we developed to predict transcription units (TrUC). The transcription unit predictions and other evidence were combined to produce gene annotations using EuGene software [12] trained for *Paramecium*. Annotations obtained for *P. tetraurelia* were used as evidence to improve the annotation of other *Paramecium* species. The *P. tetraurelia* RNA-Seq samples were also analyzed for differential gene expression during the sexual cycle of autogamy, generating an improved transcriptome resource.

## Results and Discussion

### Transcription units

**Fig. 1:**
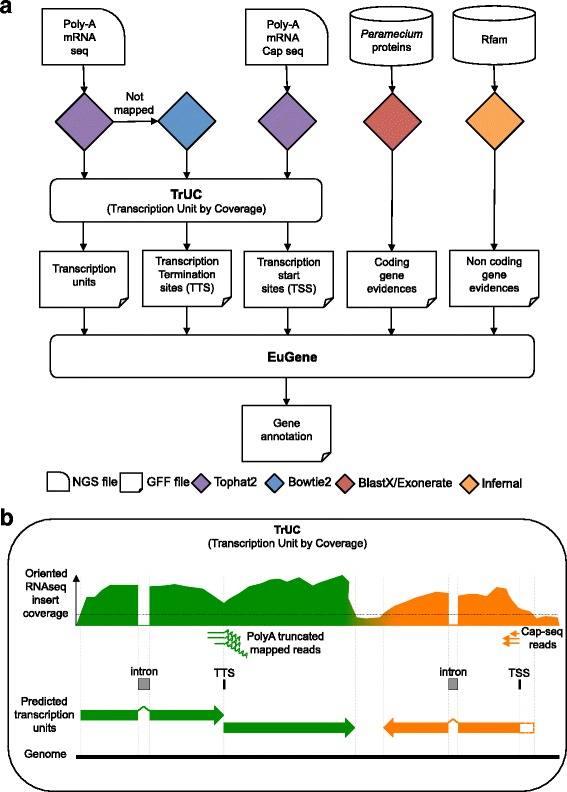
Gene annotation strategy. **a** Overview of the workflow. EuGene software, using a Paramecium-trained matrix, combines (i) transcription unit predictions, (ii) TSS predicted positions, (iii) TTS predicted positions, (iv) *Paramecium* predicted proteins mapped on the reference genome using BLASTX then Exonerate, and (v) non-coding gene predictions obtained using the Rfam database. **b** Schema of the TrUC pipeline. TrUC is able to predict transcription units, TSS and TTS positions. To achieve this, the software uses oriented polyA^+^ mRNA-Seq and Cap-Seq data. The upper part of the schema represents RNA-Seq insert coverage of the genome. A configurable threshold (horizontal dotted line) is used to determine the edges of the transcription units. The middle of the schema shows how intron, TSS and TTS positions are predicted. The transcription units predicted by combining all of the information are shown at the bottom of the schema. The TSS and the TTS are used to refine the structure of the transcription unit predictions. This can be particularly critical in a compact genome to avoid fusing adjacent transcription units. An example is shown in orange, where the TSS is used to shorten the predicted transcription unit, removing the open box. The example in green, shows how a TTS can prevent fusion of two adjacent transcription units

Genome-guided transcription unit construction was pioneered by Denoeud et al. [13] (G-Mo.R-Se software) using short-read mapping. The widely used Cufflinks software [14] then adopted fragment alignment as part of its assembly strategy. To take into account alternative splicing, Cufflinks finds the minimal number of paths through the mapped fragments. We decided to develop our own transcription unit prediction software rather than use Cufflinks, because in our hands, Cufflinks did not always predict transcription units despite good fragment coverage in regions presenting strong evidence of protein-coding genes, probably because of overlapping UTRs in the very compact *Paramecium* genome. Our approach is conceptually similar to G-Mo.R-Se but takes into account improvements in library construction and sequencing, especially orientation information. We added detection of transcription termination sites (TTS) using the polyA signal and the optional use of Cap-Seq data to predict transcription start sites (TSS). The TrUC (TRanscription Units by Coverage) pipeline is schematized in Fig. 1. TrUC predicts TSS and TTS using the consensus position of Cap-Seq and polyA coverage, respectively. TrUC uses oriented data to predict oriented transcription units, however the software can predict introns from un-oriented RNA-Seq data, using the GT.. AG splice site consensus to determine orientation. TrUC does not consider alternative splicing since exon skipping is not found in *Paramecium* [15]. Like Cufflinks, TrUC can identify non-coding transcripts as it does not look at translation. TrUC multi-threaded Perl software is available from https://github.com/oarnaiz/TrUC.

Since not all genes are expressed at all stages of the life cycle, use of different life-cycle time points can help annotation by increasing the number of genes that are covered by RNA-Seq data. For *P. tetraurelia* transcription unit prediction, we sequenced polyA+ RNA from a time-course through the sexual process of autogamy as well as from vegetative cells. Combining all the RNA-Seq and Cap-Seq data (Additional file 1: Table S1), TrUC predicts 37, 847 transcription units greater than 300 nt in size, 12,389 TSS and 5967 transcription termination sites (TTS). We found 85% of the previously annotated *P. tetraurelia* genes [6] (hereafter called “v1” annotation) covered by a predicted transcription unit. The average size of the predicted transcription units, 1229 nt, is close to the average size of the v1 genes (Table 1; Additional file 2: Figure S1) indicating that most of the predicted transcription units correspond to one gene. However, some transcription unit predictions may be split, owing to reduced RNA-Seq coverage within the unit. Alternatively, since genes in the compact *P. tetraurelia* genome sometimes overlap, some transcription units may be fused owing to continuous RNA-Seq coverage, a problem partially overcome by use of the predicted TSS and TTS (cf. Fig. 1b). Split or fused predicted transcription units do not compromise gene annotation (cf. next section), which also takes into account protein-coding potential to define gene models.

**Table 1:** Annotation statistics

	*P tetraurelia*		*P. biaurelia*		*P. sexaurelia*		*P. caudatum*	
	V1	V2	V1	V2	V1	V2	V1	V2
Genome size (nt)	72,094,543	72,102,941	75,777,660	74,348,537	67,662,147	67,662,147	29,932,356	29,932,356
Number of scaffolds	697	697	1426	1026	230	230	274	274
N50	412,884	412,881	156,715	159,040	430,207	430,207	312,370	312,370
Genomic GC content	0.28	0.28	0.25	0.25	0.24	0.24	0.28	0.28
Gene number	39,642	41,533	39,110	40,741	34,909	36,477	18,421	18,853
Gene length (nt)	1433.8	1409.91	1460.56	1430.22	1462.39	1430.91	1449.52	1448.91
Percent coding	75	75	71	73	71	73	85	86
Inter coding distance (TGA ↔ TGA)	285.23	244.88	304.13	275.83	362.68	331.12	109.29	87.73
Inter coding distance (ATG ↔ ATG)	451.27	388	462.77	410.36	565.88	501.34	143.39	120.63
Inter coding distance (TGA ↔ ATG)	332.03	286.77	357.91	333.08	433.5	393.35	131.64	116.84
Protein Coding Gene number	39,642	40,460	39,110	40,179	34,909	36,053	18,421	18,592
CDS length (nt)	1363.32	1330.35	1375.51	1359.78	1380.08	1367.54	1386.16	1385.68
Protein Coding Gene GC content	0.3	0.3	0.26	0.26	0.26	0.26	0.29	0.29
Non-coding gene number		1073		562		424		261
Exon number	130,216	136,527	141,873	135,427	126,637	124,022	63,789	62,173
Median exon length (nt)	230	222	200	221	202	213	216	232
Mean exon length (nt)	419.01	411.3	379.19	412.21	380.44	402.57	400.3	423.13
Exon per gene	3.28	3.29	3.63	3.32	3.63	3.4	3.46	3.3
Intron number	90,282	94,711	102,763	94,686	91,728	87,545	45,368	43,320
Median intron length (nt)	25	25	26	25	26	25	22	22
Mean intron length (nt)	25.14	25.31	32.37	25.82	31.32	25.91	25.72	23.3
Number of introns >40 nt	38	720	12,817	1366	9718	1218	2425	720

The genome assembly and v1 annotation of *P. tetraurelia* were published in [6]. The v2 annotation used the same assembly after polishing with Illumina reads, reported in [37]. The v1 genome assembly and annotation of *P. biaurelia* and *P. sexaurelia* were published in [7] and the v1 genome assembly and annotation of *P. caudatum* were published in [8]. The v2 annotations are those obtained in the present study. In the case of *P. biaurelia*, the reference genome was filtered to remove scaffolds of obvious bacterial origin before v2 annotation. All annotations are integrated into ParameciumDB and available for download as GFF3 files

A total of 85,236 introns were identified in the predicted transcription units, corresponding to a mean of 2.25 introns per transcription unit, very close to the 2.3 introns per gene previously reported for *P. tetraurelia* [6, 15].

### Gene annotation

Accurate, user-friendly gene annotation tools for eukaryotes, such as AUGUSTUS [16], would require code modifications to correctly identify tiny introns [17]. Indeed, ~ 98% of *P. tetraurelia* introns are 20–30 nt in size, with a mean of 25 nt. A handful of significantly larger introns, ~ 80 nt in size, contain snoRNAs [18]. We therefore decided to train the highly configurable EuGene annotation software [12] for *Paramecium*, using gene models completely confirmed by RNA-Seq coverage (see Methods).

Table 1 compares gene annotations predicted by EuGene for *P. tetraurelia*, using the transcription units assembled with TrUC and other lines of evidence (labeled ‘v2’), with the v1 gene annotation for this species [6], long considered a gold standard for ciliate gene annotation. The statistical differences are slight, aside from the fact that the v1 statistics do not include any non-coding gene predictions. The v2 annotation contains about 800 more protein-coding gene models, probably because of the greater quantity of transcript evidence allowing prediction of short genes (the average CDS length is slightly smaller in v2 annotation). To determine sensitivity, a set of 1690 manually curated genes was constituted (available from ParameciumDB [19]). We found 95% of the gene structures (excluding UTRs) and 99% of the introns to be correctly annotated.

In order to analyze the impact of the Cap-Seq data, we ran the same gene annotation pipeline for *P. tetraurelia* without any Cap-Seq data and compared the two sets of gene models. We found 91.8% of the protein-coding gene models to be identical. Among the 3293 gene models that were different, there were 649 cases where addition of the Cap-Seq data split one gene model into two gene models. In most of the remaining cases (2149), the coding sequence was changed. The Cap-Seq data we generated thus have a modest but significant impact on the gene annotation. Knowledge of TSS for the *P tetraurelia* model will be particularly valuable for functional studies. We looked for possible alternative TSS by evaluating whether adjacent TSSs fall within the same gene. In >99% of the cases, adjacent TSS were in different genes. In only 29 cases could we find adjacent TSS in the same gene. We consider it likely that they represent technical noise given their occurrence mainly in highly expressed genes (Additional file 1: Table S2) but we cannot exclude biological significance. We conclude that alternative TSS are extremely rare in *Paramecium*.

**Fig. 2:**
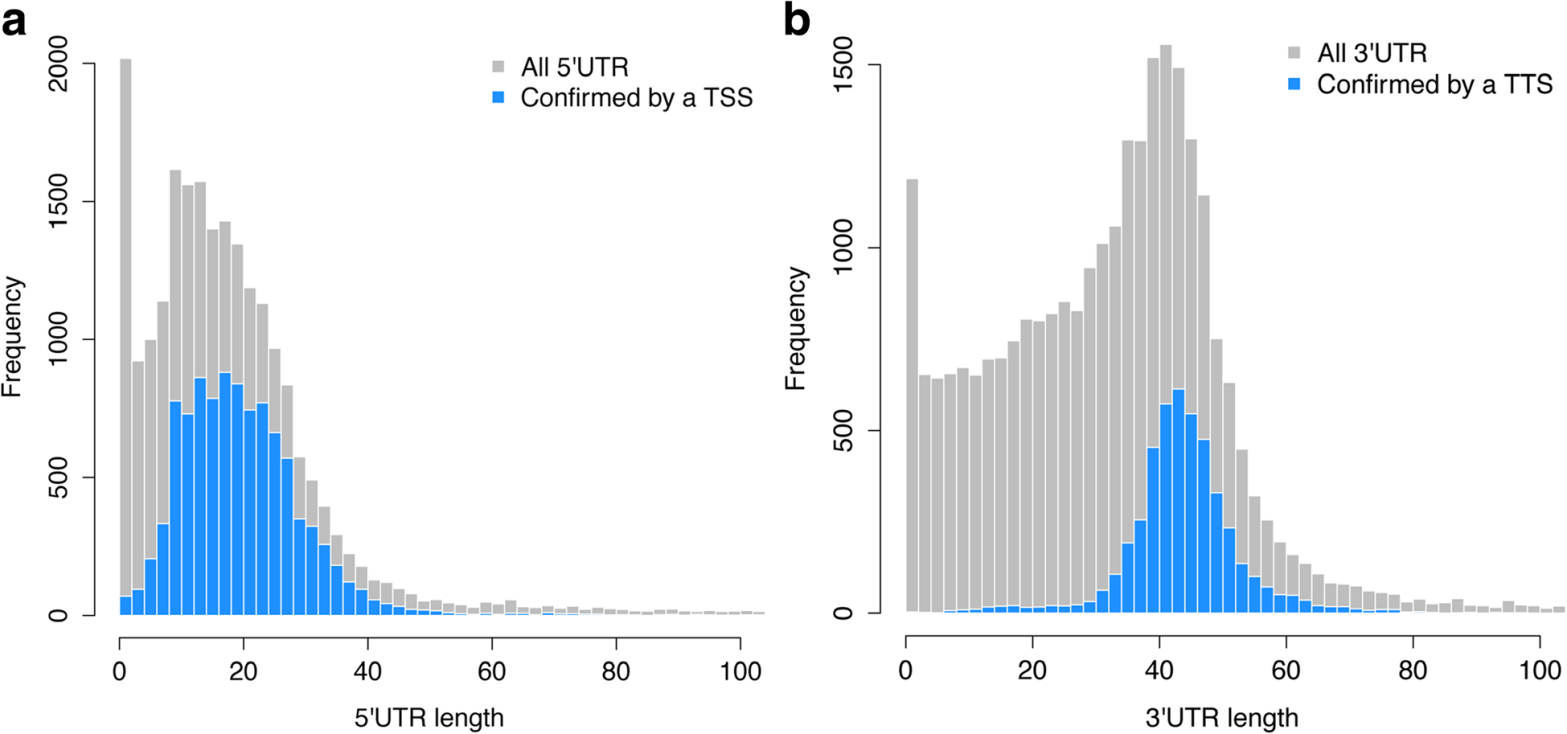
Size distribution of *P. tetraurelia* 5′ and 3′ UTRs. **a** Histograms of the distribution of predicted 5′ UTR sizes (gray) and 5′ UTR sizes confirmed by a TSS prediction using Cap-Seq data. **b** Histograms of the distribution of predicted 3′ UTR sizes (gray) and 3′ UTR sizes confirmed by a TTS prediction using the polyA^+^ RNA-Seq read pairs. The Cap-seq confirmed 5′ UTRs have a mean size of 21.7 nt and a median size of 19 nt. The 3′ UTRs predicted from polyA tracts have a mean size of 44.6 nt and a median size of 44 nt

The size distribution of 10,087 5′ UTRs that could be confidently predicted from the Cap-Seq data, shown in Fig. 2, confirms the unusually small size of 5’UTRs in *Paramecium* (mean 21.7 nt, median 19 nt), much smaller than in animals and fungi (100–200 nt average, [20]) or the ciliate *Tetrahymena thermophila* (*n* = 4149, mean = 95.6 nt, median = 88 nt) [21]. The 4641 3′ UTRs predicted from polyA tracts present in the RNA-Seq data (Fig. 2) display a nearly Gaussian distribution (mean = 44.6 nt, median = 44 nt) and are much smaller than in animals and fungi (200–1000 nt average, [20]) or *Tetrahymena thermophila* (*n* = 1290, mean = 231 nt, median = 163 nt, [21]).

**Fig. 3:**
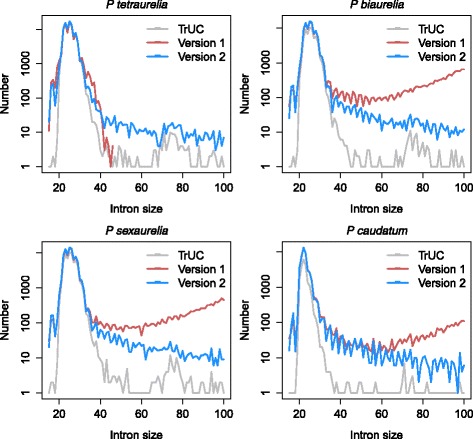
Intron size distribution. The graphs show the number of introns as a function of their size for *Paramecium tetraurelia, biaurelia, sexaurelia* and *caudatum*. For each species, 3 curves are superimposed: introns predicted by TrUC (*gray*), introns in the v1 annotations (*red*) and introns in the v2 annotations (*blue*). The v2 introns are the subset of TrUC introns in gene models and additional introns predicted by Eugene without RNA-Seq support. The *P. tetraurelia* v1 introns were intentionally filtered when greater than 45 nt [6]. In all cases, the great majority of introns are smaller than 40 nt. In v1 annotations (except *P. tetraurelia*), there are more large introns than in TrUC or v2 annotations. Since the RNA-Seq (TrUC predictions) are expected to show the true distribution of intron sizes, we are more confident in the v2 annotations

The *Paramecium-*trained annotation pipeline was used to annotate *P. biaurelia, P. sexaurelia* and *P. caudatum* genomes, using the *P. tetraurelia* v2 predicted proteins as evidence as well as the unoriented RNA-Seq data previously used for the published annotation of these species [7, 8]. In all 4 species, we observe ~2.3 introns/gene and the same median intron size in v1 and v2 annotations (Table 1). However, the average intron size is larger in the published *P. biaurelia*, *P. sexaurelia* and – to a lesser extent – *P. caudatum* v1 annotations than in the corresponding v2 annotations (Table 1). This is owing to the prediction of significant numbers of introns larger than 40 nt in the published v1 annotations (Table 1, Fig. 3). Far fewer questionable “large” introns are found in the v2 annotations (10%, 12.5% and 30% with respect to the v1 annotations for *biaurelia*, *sexaurelia* and *caudatum*, respectively). This is because the v2 annotations integrate TrUC intron predictions. Most of these “large” introns are likely to be incorrect and as a consequence, so are the thousands of gene models that contain them. The difference between v1 and v2 annotations is less pronounced for *P. caudatum,* which has the smallest introns so far reported for the *Paramecium* genus (median size 22 nt, average size 23 nt). We conclude that TrUC predictions improve *Paramecium* gene models not only by predicting TSS and TTS if Cap-Seq and oriented mRNA-Seq data are available, but also thanks to the intron predictions, which can be made even from unoriented RNA-Seq data.

*Paramecium* genomes are intron-rich, an ancestral property of eukaryotes [22]. Not only is intron size very small, but no exon-skipping has been observed [15]. These properties helped discover translational control of eukaryotic intron splicing [15]. In *Paramecium*, splice site signals are weak, presumably because of the mutational burden, and splicing is not very accurate. The nonsense-mediated decay (NMD) pathway [23] cleans up the mess on the pioneer round of translation, provided that there is a Premature Termination Codon (PTC) in the poorly spliced transcript. That this is the case is easily visualized in *Paramecium*, thanks to the unique TGA stop codon, by comparing the frequency of introns that do or do not contain a STOP codon in phase with the upstream exon, as a function of their size modulus 3. As shown in (Additional file 2: Figure S2), stopless introns that are 3n in size are counter-selected in all 4 species: these introns cannot give rise to a PTC if retained in the transcript so are potentially deleterious. The observed deficit of 3n stopless introns in all 4 species (Additional file 2: Figure S2) validates the annotation and extends previous observation of translational control of intron splicing [15] to other *P. aurelia* species and to *P. caudatum*.

### Differential gene expression

In *Paramecium*, the sexual cycle encompasses meiosis, fertilization and development of a new MAC. The latter process involves programmed elimination of at least 25% of the germline DNA. Custom microarrays were previously used to characterize differential gene expression during autogamy (auto-fertilization) in *P. tetraurelia* [9]. We now report use of the mRNA-Seq samples for differential gene expression. Since cells enter autogamy from a fixed point in the vegetative cell cycle [24], which is not synchronized in our cell cultures, there is an asynchrony of at least 5 h in the samples. Therefore, cytology data (Additional file 2: Figure S3) and gene expression levels were used to cluster samples into 6 stages: vegetative (Veg, *n* = 2), meiosis (Mei, *n* = 2), fragmentation (Frg, *n* = 3), early development (Dev1, *n* = 2), intermediate development (Dev2 and Dev3, *n* = 4) and late development (Dev4, *n* = 2) (see Methods, Additional file 1: Table S1 and Additional file 2: Figure S3). These stages are equivalent to those used previously with microarrays [9], with the addition of one later stage, Dev4.

For analysis of differential gene expression (DGE) we first counted mapped RNA-Seq fragments for each v2 gene model (see Methods). We found 99.8% of the mapped fragments in the sense orientation and 0.2% in the anti-sense (AS) orientation (see Additional file 2: Figure S4). This low level of AS transcription might reflect biological noise or pervasive transcription [25], especially as pervasive non-coding transcription is required for genome rearrangements in *Paramecium* [26, 27]. We cannot formally exclude errors in strand-specificity during construction of the sequencing libraries [28]. An intriguing possibility is regulation of gene expression by AS transcripts at specific loci. Higher coverage or long reads will be needed to interpret the AS transcription we have detected.

The DGE analysis was performed with the sense fragment counts and DESeq2 software. Requiring an adjusted *p*-value <0.01 and a fold-change of 2 in expression, we identified 17,190 genes whose expression varied during autogamy. We separated these genes into induced (*n* = 8220) or repressed (*n* = 8970) (cf. Methods), and then used hierarchical clustering to visualize the induced or repressed genes and to define 6 clusters: ‘Early peak’, ‘Intermediate peak’, ‘Late peak’, Late induction’, ‘Early repression’, ‘Late repression’ (Additional file 2: Figures S5 and S6).

To validate the clusters, we used published genes involved in autogamy and some negative control genes not involved in autogamy (Additional file 1: Table S3). All of the genes known to be induced during autogamy were classified in an appropriate RNA-Seq induced cluster, although 4 of the 26 genes had not been found in the microarray clusters. Genes known to be expressed during meiosis in *Paramecium* (*SPO11*, *SPT5m*, *DCL2*, *DCL3*, *NOWA1*, *NOWA2, LIG4a* and *XRCC4*) [29–33] are found in the Early peak. Only one of the 12 negative control genes was induced during autogamy: *PTIWI14*, characterized as a component of the vegetative siRNA pathway [34], appears in the Late induction cluster. We also compared the distribution of the genes in the microarray clusters with respect to the RNA-Seq clusters (Table 2). The microarray resource was of good quality and essentially all of the genes in the microarray clusters are found in the RNA-Seq clusters. The RNA-Seq approach is more powerful and allows more genes to be identified as differentially expressed during autogamy. A modest qualitative difference between microarray and RNA-Seq classification concerns the microarray ‘Intermediate induction’ cluster. The genes in this cluster are found in either the RNA-Seq ‘Intermediate peak’ or the RNA-Seq ‘Late induction’ clusters. This may be a consequence of the additional late time points in the RNA-Seq experiment (Dev4) which change the gene expression profiles being clustered.

**Table 2:** Differential Gene Expression

	Early peak	Intermediate peak	Late peak	Late induction	Early repression	Late repression	none
Early peak	333	17	0	1	0	8	1
Early induction	49	33	1	9	0	0	0
Intermediate induction	5	315	17	211	0	0	1
Late induction	0	7	0	28	1	0	0
Early repression	0	0	0	1	209	30	4
Late repression	28	0	0	0	1	1022	3
none	1315	1398	395	2937	3986	3072	20,043

Distribution of differentially expressed (DE) genes in RNA-Seq clusters (columns) and microarray clusters [9] (rows). Overall, the two analyses provide similar results, but the RNA-Seq approach detects many more DE genes. Essentially all microarray DE genes were found by RNA-Seq. The main qualitative difference is that the genes in the microarray Intermediate induction cluster are now found in one or the other of 2 RNA-Seq clusters: Intermediate peak or Late induction. See Methods for details of the analysis

To estimate how well RNA-Seq data can discriminate the ~12,000 pairs of paralogs created by the recent WGD (~83% nucleotide identity on average), we computed the number of identical stretches of 100 nt shared by each pair. We estimate that >97% of the paralog pairs are devoid of common 100 nt stretches over >90% of their length and should therefore be well-discriminated in the present analysis. This is likely to be an underestimate, since the procedure uses mapping of pairs of 100 nt reads, not of single 100 nt reads. Therefore, in theory, RNA-Seq should better discriminate the paralogs than the previous microarray data [9]. In addition, RNA-Seq has greater dynamic range and sensitivity than microarray technology ([35] and our data), which can also contribute to good paralog discrimination. The *P tetraurelia* microarrays involved hybridization of 6 probes (50 nt) per gene designed to optimize chances of discriminating the paralogs. However, it is difficult to evaluate the extent of microarray cross-hybridization. In order to compare the discrimination of WGD paralogs by the microarray and RNA-Seq methods, we calculated paralog expression level divergence (Additional file 2: Figure S7). Paralogs are more sensitively discriminated by RNA-Seq because of its greater dynamic range (Additional file 2: Figure S7a, b). When the paralogs share high nucleotide identity (with the greatest risk of cross-hybridization), there is little difference between their expression levels in the microarray data, but the same is true of the RNA-Seq data (Additional file 2: Figure S7a–d). We propose two, non-exclusive, explanations to account for this observation, the first technical, the second biological. The first explanation is that RNA-Seq cannot discriminate highly similar paralogs because reads are mapped randomly between 2 equivalent loci, which reduces the difference in the measured expression level of such loci. The second explanation is that the paralogs sharing high nucleotide identity probably tend to have similar expression levels. This could result from strong selective pressure on highly expressed genes [10] or from gene conversion between WGD paralogs, frequently observed for *P. aurelia* species [7]. Gene conversion leads to increased GC content and high nucleotide identity between paralogs, and can extend to promotor regions depending on the recombination breakpoint. Indeed, expression level and GC content are correlated with the nucleotide identity of *P. tetraurelia* paralogs (Additional file 2: Figure S7e, f).

We also looked at whether genes duplicated at the recent WGD have kept the same expression profiles. First, we removed paralogs that differ in length by more than 10%, a filter that removes potential pseudogenes (*n* = 10,323; 85% of the paralog pairs are retained after this stringent filtering, *P. tetraurelia* v2 annotation). For 22% of the pairs, we found both paralogs in the same autogamy cluster, and for 38%, neither paralog was differentially expressed during autogamy. Interestingly, we found 31.5% of the pairs had one paralog in an autogamy cluster and one paralog not differentially expressed. This might reflect an early stage of pseudogenization, shown to begin with changes in expression level [11], probably via mutations in promoter sequences. We also found 8.5% of pairs with paralogs in different autogamy clusters, a possible indication of neo- or sub-functionalization. The important point is that finding paralogs with different expression profiles, irrespective of the origin of the difference, is an indication that the paralogs are well-discriminated by the RNA-Seq data.

A qualitative picture of the biological processes turned on during autogamy can be obtained using Gene Ontology (GO) terms [36], with the caveat that *Paramecium* functional annotation has not been curated. We first made a high-confidence gene set by requiring a fold-change of 4 during autogamy (7065 genes). We then used GO Biological Process terms associated with protein domains (cf. Methods) to make word clouds (Additional file 2: Figure S8). The Early peak (Additional file 2: Figure S8a) covers meiosis and fertilization and is enriched in appropriate terms: *DNA*, *repair*, *chromosome*, *mismatch*, *condensation, homologous*, *recombination*, *Okazaki*, *replication*, *chromatid*, *cohesion*. The Intermediate peak (Additional file 2: Figure S8b) corresponds to development of the new MAC involving chromatin remodeling and shows enrichment in *repair, chromatin, DNA*, *methylation*, and *chromosome*. Many biological processes are activated in the Late peak and Late induction clusters so that the only over-represented informative words relate to signal transduction (*GTPase, signal, transduction, phosphorylation, inositol)* and membrane trafficking (*vesicle-mediated, autophagy)* (Additional file 2: Figure S8c, d). The cluster of genes that are turned off when cells leave vegetative growth and enter the sexual cycle (Early repression, Additional file 2: Figure S8e) is enriched for words that refer to translation and cellular homeostasis (*rRNA, redox, homeostasis, ribosome, oxido-reduction, translation, pseudouridine)*. For the Late repression cluster (Additional file 2: Figure S8f), the only informative words refer to *microtubule-based, movement* and *phosphorylation, dephosphorylation.*

The gene expression atlas is provided as a table (Additional file 3: Table S4).

## Conclusions

Plummeting genome sequencing costs and rising interest in *Paramecium* species for studies of genome evolution in unicellular eukaryotes, prompted us to build a new pipeline for gene annotation that takes into account specificities of the genus, in particular high gene density and stereotyped small intron size. This has been achieved with new software to predict transcription units (TrUC) and specific training of the highly configurable EuGene gene annotation software. High quality gene annotations will be important for future comparative and functional genomics analyses of *Paramecium* species. The mRNA-Seq data used to predict *P. tetraurelia* transcription units for the gene annotation enabled us to generate an improved gene expression atlas and carry out differential gene expression analysis of the sexual cycle of autogamy that is more complete than previously possible with microarrays.

## Methods

### RNA samples and mRNA sequencing

Three time-course experiments for the sexual process of autogamy of *P. tetraurelia* wild-type strain 51 were used. Some of the samples had previously been used for microarray experiments [9] (see Additional file 1: Table S1). Total RNA was Trizol-extracted as previously described [9]. PolyA^+^ RNA was extracted from each sample using the FastTrack MAG mRNA isolation kit (Thermo Fisher Scientific) following the manufacturer’s instructions. Strand-oriented Illumina libraries were made with reagents from the Illumina TruSeq Small RNA library preparation kit using an adapted protocol. First, RNA was fragmented by incubation for 4 min at 94 °C with New England Biolabs’ Mg2+ solution, yielding fragments with an average size of ~260 nt. The RNA was purified using RNeasy columns (Qiagen) followed by treatment with antarctic phosphatase and polynucleotide kinase (New England Biolabs) and another purification on RNeasy columns. Illumina adapter ligation and RT-PCR was done essentially following the Illumina protocol, except that for the final library purification step AMPure beads were used (Beckman Coulter). HiSeq paired-end sequencing was performed on the samples, yielding at least 2 × 30 million reads, 100 nt in length, for each of the samples.

### Cap-Seq

Five samples, representing 3 time points (Veg, T0 and T11; [9]) were used for 5′ Transcription Start Site (TSS) mapping. Purified polyA^+^ RNA was dephosphorylated using Calf Intestine Phosphatase (CIP) prior to 5′ cap removal with Tobacco Acid Pyrophosphatase (TAP), using a FirstChoice RLM-RACE kit (Life Technologies). Illumina 5′ adaptors were ligated to the 5′ monophosphate ends generated specifically at TSSs, followed by RNA fragmentation by incubation for 4 min at 94 °C with New England Biolabs’ Mg^2+^ solution. This yielded an average fragment size of 260 nt. Following CIP treatment to convert the 3′ monophosphate ends generated by RNA fragmentation to 3’OH ends, Illumina 3′ adapters were ligated to the fragments. The libraries were subjected to RT-PCR amplification (18–20 cycles) before Illumina HiSeq paired-end sequencing that yielded approximately 2 × 13 million reads of 100 nt per sample. Every step in TSS library preparation ended with purification using RNAeasy columns (Qiagen) or phenol/chloroform extraction and isopropanol precipitation. The final PCR amplification products were purified using AMPure beads (Beckman Coulter) before sequencing.

### Transcription unit determination

We developed the multi-threaded Perl software TrUC (Transcription Units by Coverage), dependent on the Bio::DB::Sam module. The software is organized in 3 independent modules (Fig 1). The TSS module uses Cap-Seq data, which need not be paired-end, to predict transcription start sites. The predicted TSS is the position with the highest Cap-Seq coverage in the interval defined by the size of the fragments. The TTS module uses oriented paired-end mRNA-seq reads. If one of the reads in the pair maps partially on the reference genome and ends in polyA, then the insert is used to specify a transcription termination site. The predicted TTS is the position with the highest polyA coverage in the interval defined by the size of the fragments. The transcript module takes paired-end TopHat2 mapping (BAM files; [14]) and optionally, the output of the TSS and TTS modules, to predict transcription units including intron positions, based on fragment coverage. TrUC was run with the following parameters for *P. tetraurelia* annotation:

truc TSS -min_coverage 15 -nb_replicates 2 -min_score 500; truc TTS -min_coverage 5 -nb_replicates 2 -min_score 10 -nb_min_A 5; truc transcript -min_splicing_rate 0.7 -no_overlap -min_coverage 15 \

-intron_consensus -min_intron_length 15 -max_intron_length 100 \
-min_intron_coverage 15 -min_length 300 -min_score 45 -tss [truc TSS GFF3 output file] -tts [truc TTS GFF3 output file].

For *P. biaurelia, P. sexaurelia and P. caudatum,* TrUC was used with unoriented RNA-Seq data reported in [7, 8] (Accessions PRJNA268243, PRJNA268244 and PRJNA268245, respectively), to predict introns, with the following parameters:

- truc transcript -not_stranded -min_splicing_rate 0.7 -min_coverage 10 -intron_consensus -min_intron_length 10 \ -max_intron_length 100 -min_intron_coverage 3 -min_length 300 -min_score 10.
- TrUC is distributed under a GNU GPL v3 license at https://github.com/oarnaiz/TrUC.

### Gene annotation

The workflow for gene annotation is schematized in Fig. 1. EuGene software [12] was used to predict gene structure. EuGene was trained with 1597 curated *Paramecium tetraurelia* genes to generate a prediction matrix that takes into account the unusually small size of *Paramecium* introns [15]. The prediction matrix is available from http://paramecium.i2bc.paris-saclay.fr/download. The evidence sets used for annotation of the 4 *Paramecium* genomes are available on request.

### Comparative genomics

UTR lengths for *Tetrahymena thermophila* were calculated using the June 2014 annotation available at http://www.ciliate.org/system/downloads/T_thermophila_June2014.gff3.

### Differential gene expression

Paired-end RNA-Seq reads were mapped to the reference *P. tetraurelia* strain 51 genome [37] using TopHat2 (v2.0.12, −−mate-inner-dist 50 --mate-std.-dev 100 --min-intron-length 15 --max-intron-length 100 --coverage-search --keep-fasta-order --library-type fr-secondstrand --min-coverage-intron 15 --max-coverage-intron 100 --min-segment-intron 15 --max-segment-intron 100 --max-multihits 1 --read-mismatches 1 --max-deletion-length 1 --max-insertion-length 1). Raw fragment counts for the genes in each sample, determined using htseq-count (v0.6.0 --stranded = yes --mode = intersection-nonempty) on filtered BAM files (samtools v0.1.18 samtools view -q 30), were used as input for DESeq2 (v1.4.1) [38], an R Bioconductor package, which normalizes the fragment counts, calculates the dispersion in the data using the biological replicates, and then determines differential gene expression using negative binomial linear models. The samples were grouped into biological replicates (Veg, Mei, Frg, Dev1, Dev2_3, Dev4) using the cytology data and clustering of the sample normalized counts with a distance matrix (see Additional file 2: Figures S3 and S4 sample dendrogram; see Additional file 1: Table S1). We considered genes to be differentially expressed during autogamy if at least one contrast between Veg and any point in the autogamy time course had an adjusted *p*-value smaller than 0.01 and a fold-change (FC) of expression greater than 2. We filtered out genes if there was not at least one time point with more than 20 normalized counts. The genes were classed as induced (FC > 2) or repressed (FC < ½) before hierarchical clustering.

### GO term enrichment and word cloud visualization

To gain qualitative appreciation of the processes associated with the groups of co-expressed genes, we focused on a subset of differentially expressed genes with a fold-change >4 (and an adjusted *p*-value <0.01). GO biological process terms were electronically inferred using InterProScan (v5.7.48) domain annotation of the corresponding proteins. If more than one protein domain was associated with a protein, the domain with the lowest InterProScan *P*-value was retained. All words in the terms were counted for all the protein-coding genes in the genome and for the protein-coding genes in each co-expression group. After removing non-discriminatory words (“protein”, “process”, “domain” and the “stopwords” defined by the wordcloud R package, v. 2.5), a Fisher exact test was used to determine the word enrichment ratio (*p*-value <0.05) in each co-expression group with respect to the word frequency for the whole genome. A score determined for each word (score = log_2_(*p*-value^−1^)) was used as weight to draw each word cloud (R wordcloud v2.5). The protein domains and GO terms used for this analysis can be found in the gene expression atlas (Additional file 3: Table S4).

## Additional files

Additional file 1: **Table S1.** RNA sequencing. **Table S2.** Genes with potential alternative TSS. **Table S3.** Differential expression of genes with known autogamy expression profiles. (XLSX 38 kb)

Additional file 2: **Figure S1.** Comparison of the sizes of *P. tetraurelia* transcription units and genes. **Figure S2.** Intron size distributions. **Figure S3.** Autogamy time-course experiments. **Figure S4.** Anti-sense transcription. **Figure S5.** Hierarchical clustering of differentially expressed genes. **Figure S6.** Autogamy co-expression clusters. **Figure S7.** Paralog discrimination by microarrays and RNA-Seq. **Figure S8.** Word cloud analysis of biological processes in clusters. (PDF 8271 kb)

Additional file 3: Gene expression atlas. All *P. tetraurelia* v2 genes (‘ID’) with their normalized RNA-Seq counts (last 15 columns, sample labels as in Additional file 2: Table S1) are given. The mean value for biological replicates are given in the columns VEG, MEI, FRG, DEV1, DEV2/3, DEV4. The ‘*P*-value’ ‘Significant’ and ‘Expression profile’ refer to the differential gene expression analysis (cf. Methods). ‘Note’ is the description of the best SwissProt BLASTP match. The GO ID and GO description were inferred electronically using InterProScan. The Biological Process GO term associated with the highest scoring protein domain is given. (TSV 12379 kb)

## Abbreviations

AS: Anti-sense
DE: Differentially Expressed
DGE: Differential Gene Expression
GFF3: Generic Feature Format, version 3
GO: Gene Ontology
MAC: Macronucleus
MIC: Micronucleus
PTC: Premature Termination Codon
TrUC: Transcription Units by Coverage
TSS: Transcription Start Site
TTS: Transcription Termination Site
WGD: Whole Genome Duplication
