# Supplementary material for: Improved methods and resources for paramecium genomics: transcription units, gene annotation and gene expression
Source: BMC Genomics. 2017 Jun 26;18:483. doi: 10.1186/s12864-017-3887-z (PMC5485702; doi:10.1186/s12864-017-3887-z)
Supplement: Supplementary file 2 — Figure S1. Comparison of the sizes of P. tetraurelia transcription units and genes. Figure S2. Intron size distributions. Figure S3. Autogamy time-course experiments. Figure S4. Anti-sense transcription. Figure S5. Hierarchical clustering of differentially expressed genes. Figure S6. Autogamy co-expression clusters. Figure S7. Paralog discrimination by microarrays and RNA-Seq. Figure S8. Word cloud analysis of biological processes in clusters. (PDF 8271 kb) [file 12864_2017_3887_MOESM2_ESM.pdf]

Fig. S1. Comparison of the sizes of *P. tetraurelia* transcription units and genes. The two distributions are very similar, illustrating the good quality of the predicted transcription units. The gene sizes for this analysis are the v1 gene annotations [1].

Fig. S2. Intron size distributions. The first column shows the size distributions of all introns for each of the 4 species. The sizes are shown modulus 3 (3n introns, black; 3n+1, red; 3n+2 green). The middle column shows the same curves, but only for introns that do not contain an in-frame stop codon. The last column shows only introns that do contain an in-frame stop codon. As previously observed for *P. tetraurelia* [2], the three curves are superposed for stop-containing introns, however there are significantly fewer stopless introns of length 3n, consistent with the hypothesis that 3n stopless introns have been counter-selected during evolution.

Fig. S3. Autogamy time-course experiments. The top part of the figure shows barplots of the cytology of RNA samples that were subjected to RNA-Seq analysis and indicates how the 15 RNA-Seq samples have been grouped into 6 groups of biological replicates (Veg, Mei, Frg, Dev1, Dev2_3, Dev4) for differential expression analysis (Table S1). Different stages of autogamy are pictured beneath the barplots. The nuclear features used for classification of a population of 100 cells are indicated in colors. When vegetative cells (**V**) enter the sexual process of autogamy (i) the two genetically identical micronuclei undergo meiosis (**M**) ; (ii) 7 of the 8 haploid products are destroyed and the remaining haploid nucleus is copied by mitosis; (iii) two haploid gametic nuclei fuse to form a diploid zygotic nucleus and the maternal MAC begins to unwind taking on the appearance of a skein (**S**); (iv) the zygotic nucleus divides twice by mitosis to yield 4 products, two become new MICs and 2 develop into new MACs. The maternal MAC breaks up into about 30 fragments (**F**); (v) during development, the new MACs (**NM**) undergo endoreplication and elimination of repeated sequences and of single-copy Internal Eliminated Sequences (IES) and (vi) at the

end of MAC development, the new vegetative cycle begins with a karyonidal divison (**K**), so-called because the new MACs, still undergoing endoreplication to achieve 800n, do not divide but are distributed to the daughter cells.

Fig.S4. Anti-sense transcription. *P. tetraurelia* polyA+ mRNA-Seq data from all samples were pooled. For each *P. tetraurelia* v2 protein-coding gene model covered by more than 10 RNA-Seq fragments, the percentage in anti-sense orientation was calculated. A. Histogram of genes according to the percentage of anti-sense fragments. Genes with less than 1% anti-sense transcription = 31,165 genes (first bar of the histogram, 80% of the genes used for the analysis). B. Dotplot of gene expression level (number of mapped fragments divided by gene length) as a function of the percentage of anti-sense fragments for that gene. The vast majority of genes have no or only a few anti-sense transcripts, suggesting transcriptional noise. A few genes have a very high percentage of anti-sense fragments and probably correspond to annotation errors. Further experiments will be required to determine whether any of the anti-sense transcription we observe has functional significance. At least some of the anti-sense transcription may arise from known vegetative and zygotic genome-wide non-coding transcription necessary for genome rearrangements. [3,4]

Fig. S5. Hierarchical clustering of differentially expressed genes. The heatmaps (generated using the R 'heatmap.2' method) show all differentially expressed genes after hierarchical clustering. The colors in the heatmaps vary from dark blue to dark red according to log2 normalized gene expression level (Color key, to the right of the heatmaps). The columns of the heatmaps correspond to the RNA-Seq samples and the rows correspond to the differentially expressed genes. The gray boxes below the sample dendrogram show the biological replicates used in the differential gene expression analysis. a. Genes that are induced during autogamy. Four gene clusters were retained, indicated by the colored bars to the right of the gene dendrogram, from top to bottom: blue, Intermediate peak; red, Late peak;

purple, Early peak and green, Late induction. b. Genes that are repressed during autogamy. Two gene clusters were retained, as indicated by the solid bars to the right of the gene dendrogram: pink, Late repression; orange, Early repression.

Fig. S6. Autogamy co-expression clusters. Each graph shows a boxplot of the normalized expression (using the mean expression value of each gene across the biological replicates for each of the 6 stages of the time-course) of the genes in the cluster. The super-imposed colored curve gives the mean normalized gene expression. The colors are the same as in Fig. S4.

Fig. S7. Paralog discrimination by microarrays and RNA-Seq. Analyses involved 9402 pairs of paralogs (*P. tetraurelia* v1 annotation) issued from the recent WGD and whose lengths differ by less than 10% (to avoid possible pseudogenes or annotation errors). We define expression level divergence for each pair of paralogs as the absolute difference between log-transformed mean expression levels for Vegetative points, after normalization (comparable results were obtained using mean expression levels for each of the autogamy stages). The expression level divergence was calculated for both the previously published microarray data and the RNA-seq data. **a.** Boxplots of the paralog expression level divergence, as a function of nucleotide identity of the paralogs, for both the microarray and the RNA-Seq data. **b.** Scatter plot of the microarray (x-axis) and the RNA-seq (y-axis) paralog expression level divergence for all the pairs of paralogs. The dotted blue line is the linear model that best fits the data. The text inside the plot gives the adjusted R^2 coefficient of the linear regression ($R^2 = 0.4$, indicating a correlation) and the p-value. **c.** Stacked histogram of the absolute difference in paralog expression level divergence between microarray and RNA-seq measurements, as a function of nucleotide identity of paralog pairs, showing that the higher the nucleotide identity, the smaller the difference between RNA-seq and microarray paralog divergence measurements. **d.** Scatter plot of the microarray (x-axis) and

the RNA-seq (y-axis) paralog expression level divergence of the paralog pairs that share at least 95% nucleotide identity. The RNA-seq and microarray paralog expression level divergence measurements are no longer correlated ($R^2 = 0.015$). **e.** Stacked histogram showing that expression level of paralogs tends to increase with increased nucleotide identity between paralogs. **f.** Stacked histogram showing that GC content of paralogs tends to increase with increased nucleotide identity between paralogs.

Fig. S8. Word cloud analysis of biological processes in clusters. Word clouds were drawn for the words in GO Biological Process terms associated with the genes in each cluster, weighted for enrichment of the word (cf. Methods). **a**. Early peak. **b**. Intermediate Peak. **c**. Late peak. **d**. Late induction. **e**. Early repression. **f**. Late repression.

Literature cited in Supplementary Figure legends

1. Aury J-M, Jaillon O, Duret L, Noel B, Jubin C, Porcel BM, et al. Global trends of whole-genome duplications revealed by the ciliate Paramecium tetraurelia. Nature. 2006;444:171–8.

2. Jaillon O, Bouhouche K, Gout J-F, Aury J-M, Noel B, Saudemont B, et al. Translational control of intron splicing in eukaryotes. Nature. 2008;451:359–62.

3. Lepère G, Bétermier M, Meyer E, Duharcourt S. Maternal noncoding transcripts antagonize the targeting of DNA elimination by scanRNAs in Paramecium tetraurelia. Genes Dev. 2008;22:1501–12.

4. Maliszewska-Olejniczak K, Gruchota J, Gromadka R, Denby Wilkes C, Arnaiz O, Mathy N, et al. TFIIS-Dependent Non-coding Transcription Regulates Developmental Genome Rearrangements. PLoS Genet. 2015;11:e1005383.

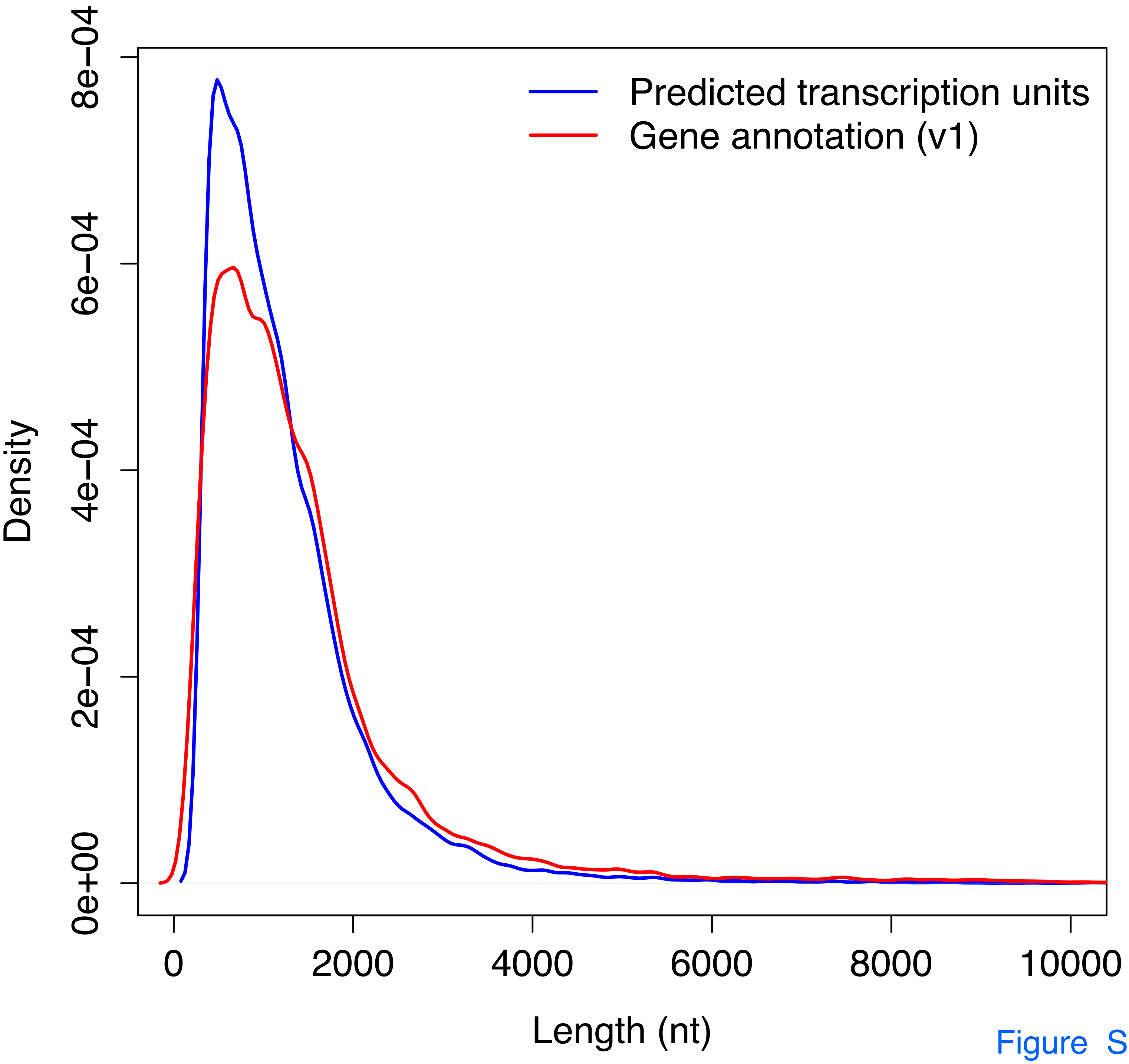

Predicted transcription units
Gene annotation (v1)
Density
0e+00
2e−04
4e−04
6e−04
8e−04
0
2000
4000
6000
8000
10000
Length (nt)

Figure S1

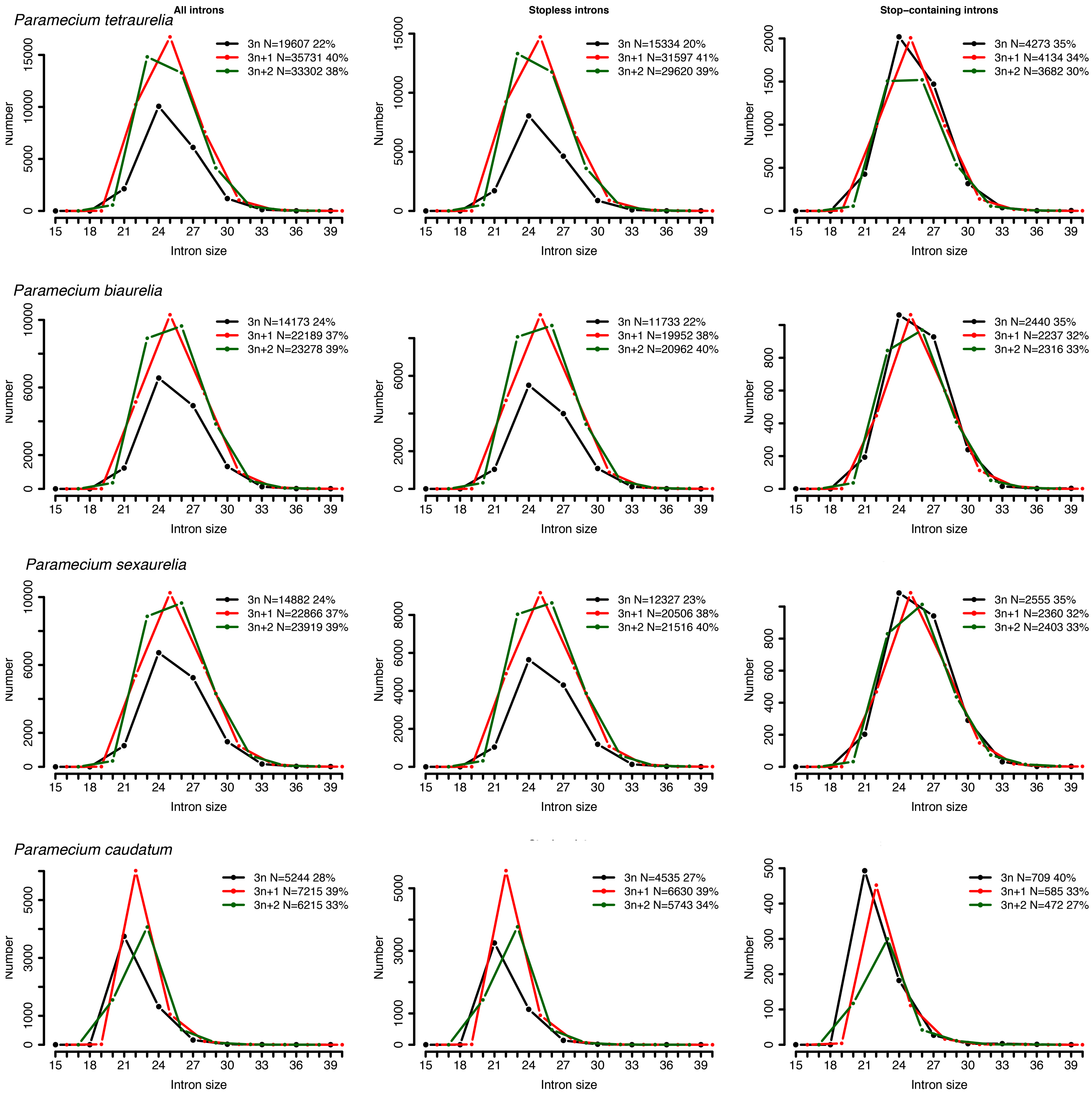
All introns
Stopless introns
Stop−containing introns
Paramecium tetraurelia
3n N=19607 22%
3n+1 N=35731 40%
3n+2 N=33302 38%
3n N=15334 20%
3n+1 N=31597 41%
3n+2 N=29620 39%
3n N=4273 35%
3n+1 N=4134 34%
3n+2 N=3682 30%
Paramecium biaurelia
3n N=14173 24%
3n+1 N=22189 37%
3n+2 N=23278 39%
3n N=11733 22%
3n+1 N=19952 38%
3n+2 N=20962 40%
3n N=2440 35%
3n+1 N=2237 32%
3n+2 N=2316 33%
Paramecium sexaurelia
3n N=14882 24%
3n+1 N=22866 37%
3n+2 N=23919 39%
3n N=12327 23%
3n+1 N=20506 38%
3n+2 N=21516 40%
3n N=2555 35%
3n+1 N=2360 32%
3n+2 N=2403 33%
Paramecium caudatum
3n N=5244 28%
3n+1 N=7215 39%
3n+2 N=6215 33%
3n N=4535 27%
3n+1 N=6630 39%
3n+2 N=5743 34%
3n N=709 40%
3n+1 N=585 33%
3n+2 N=472 27%
Number
Intron size

Figure S2

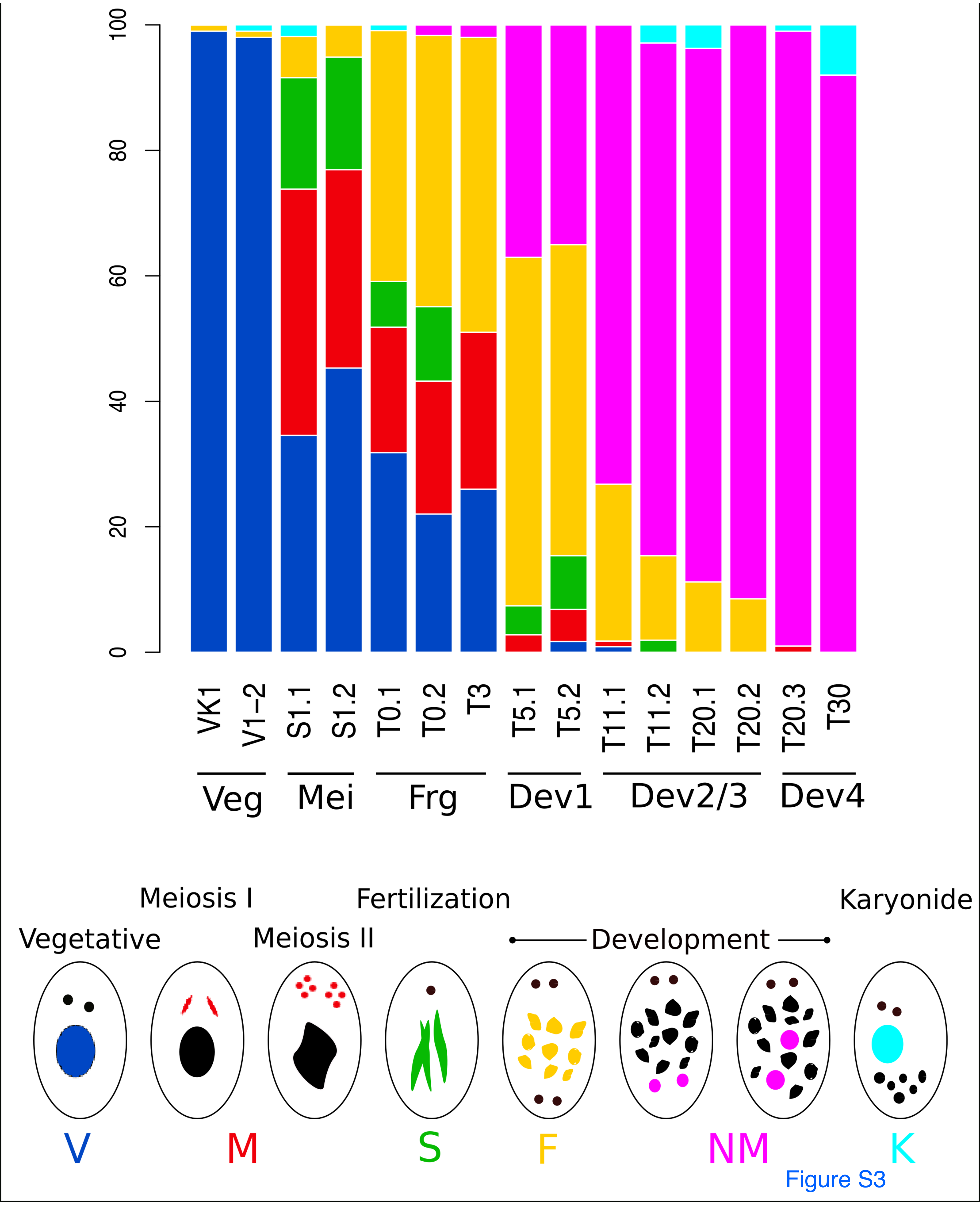

100
80
60
40
20
0
VK1
V1−2
S1.1
S1.2
T0.1
T0.2
T3
T5.1
T5.2
T11.1
T11.2
T20.1
T20.2
T20.3
T30
Veg
Mei
Frg
Dev1
Dev2/3
Dev4
Meiosis I
Fertilization
Karyonide
Vegetative
Meiosis II
Development
V
M
S
F
NM
K

Figure S3

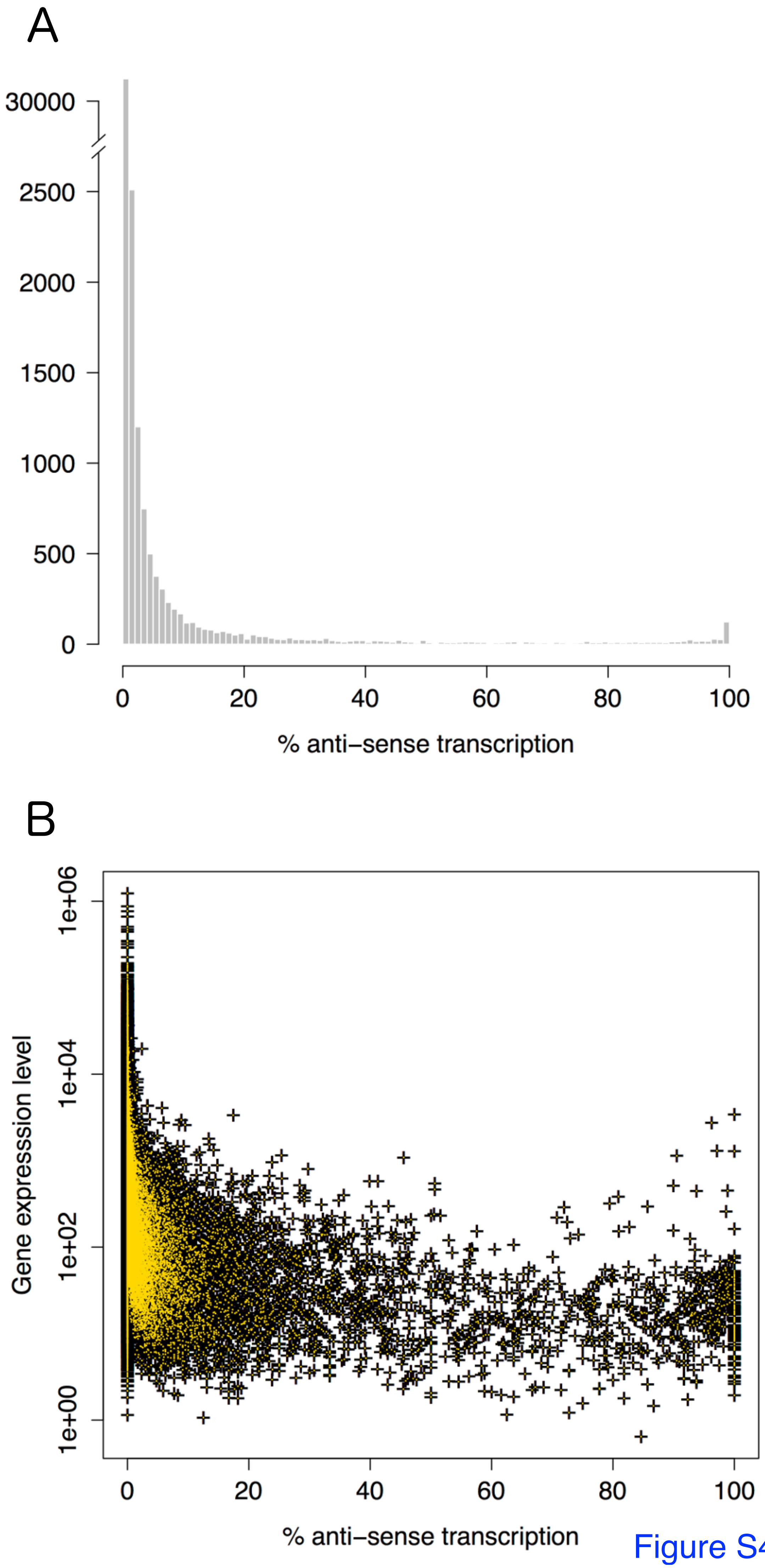
A
30000
2500
2000
1500
1000
500
0
0
20
40
60
80
100
% anti–sense transcription
B
Gene expression level
1e+06
1e+04
1e+02
1e+00
0
20
40
60
80
100
% anti–sense transcription

Figure S4

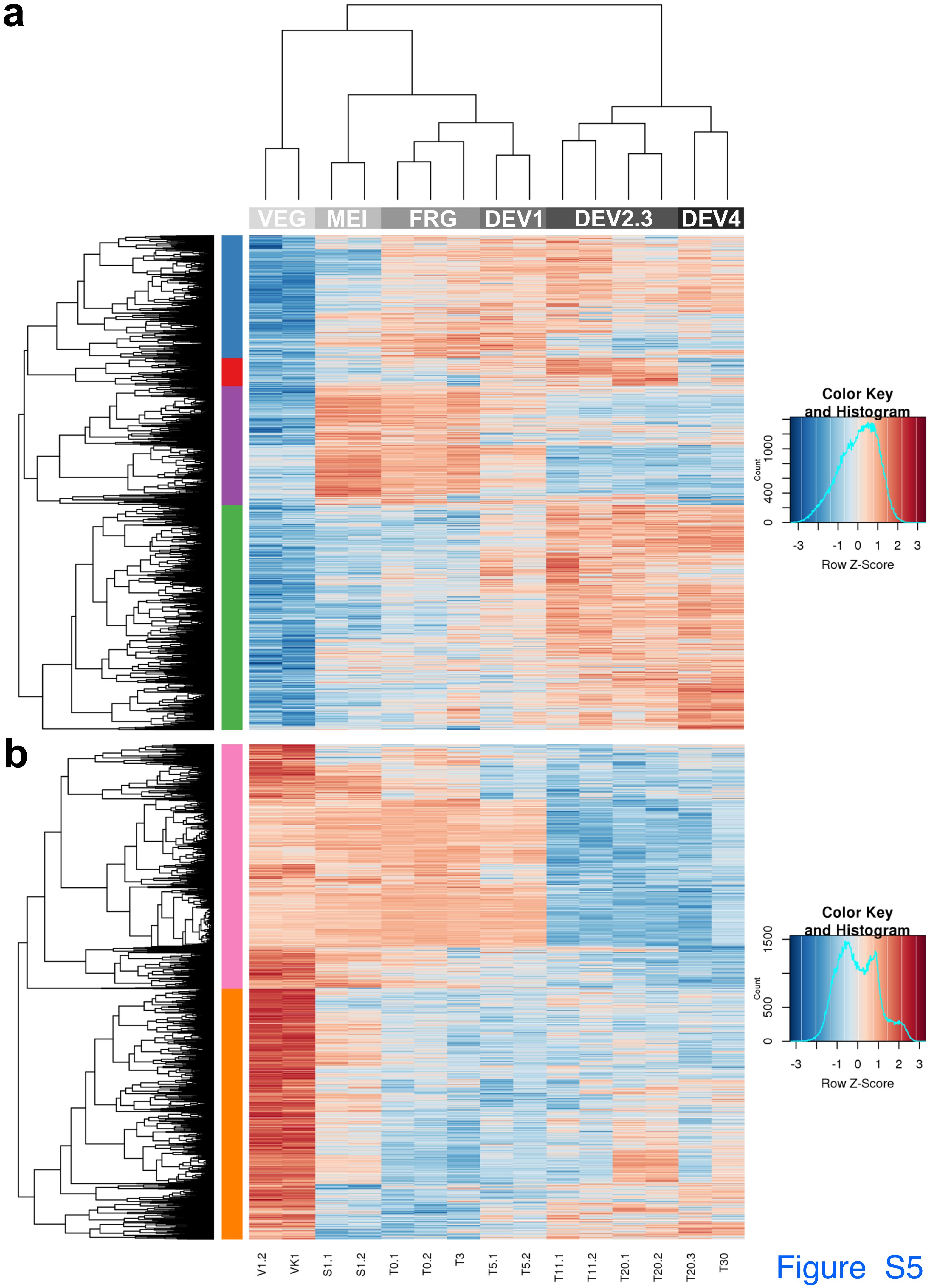
a
b
VEG
MEI
FRG
DEV1
DEV2.3
DEV4
Color Key and Histogram
Count
Row Z-Score
-3 -1 0 1 2 3
0 400 1000
0 500 1500
V1.2
VK1
S1.1
S1.2
T0.1
T0.2
T3
T5.1
T5.2
T11.1
T11.2
T20.1
T20.2
T20.3
T30

Figure S5

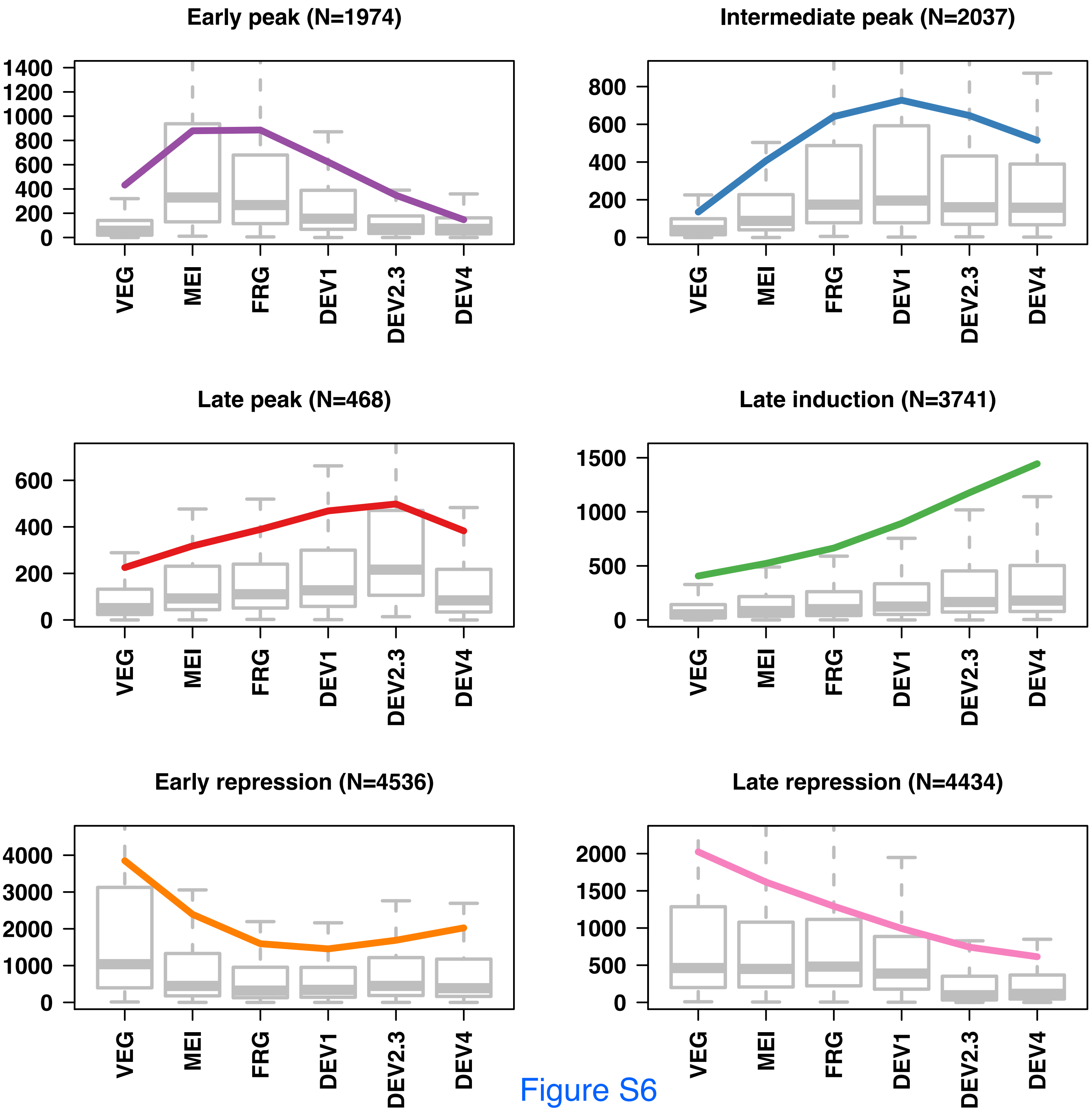

Early peak (N=1974)
1400
1200
1000
800
600
400
200
0
VEG
MEI
FRG
DEV1
DEV2.3
DEV4
Intermediate peak (N=2037)
800
600
400
200
0
VEG
MEI
FRG
DEV1
DEV2.3
DEV4
Late peak (N=468)
600
400
200
0
VEG
MEI
FRG
DEV1
DEV2.3
DEV4
Late induction (N=3741)
1500
1000
500
0
VEG
MEI
FRG
DEV1
DEV2.3
DEV4
Early repression (N=4536)
4000
3000
2000
1000
0
VEG
MEI
FRG
DEV1
DEV2.3
DEV4
Late repression (N=4434)
2000
1500
1000
500
0
VEG
MEI
FRG
DEV1
DEV2.3
DEV4

Figure S6

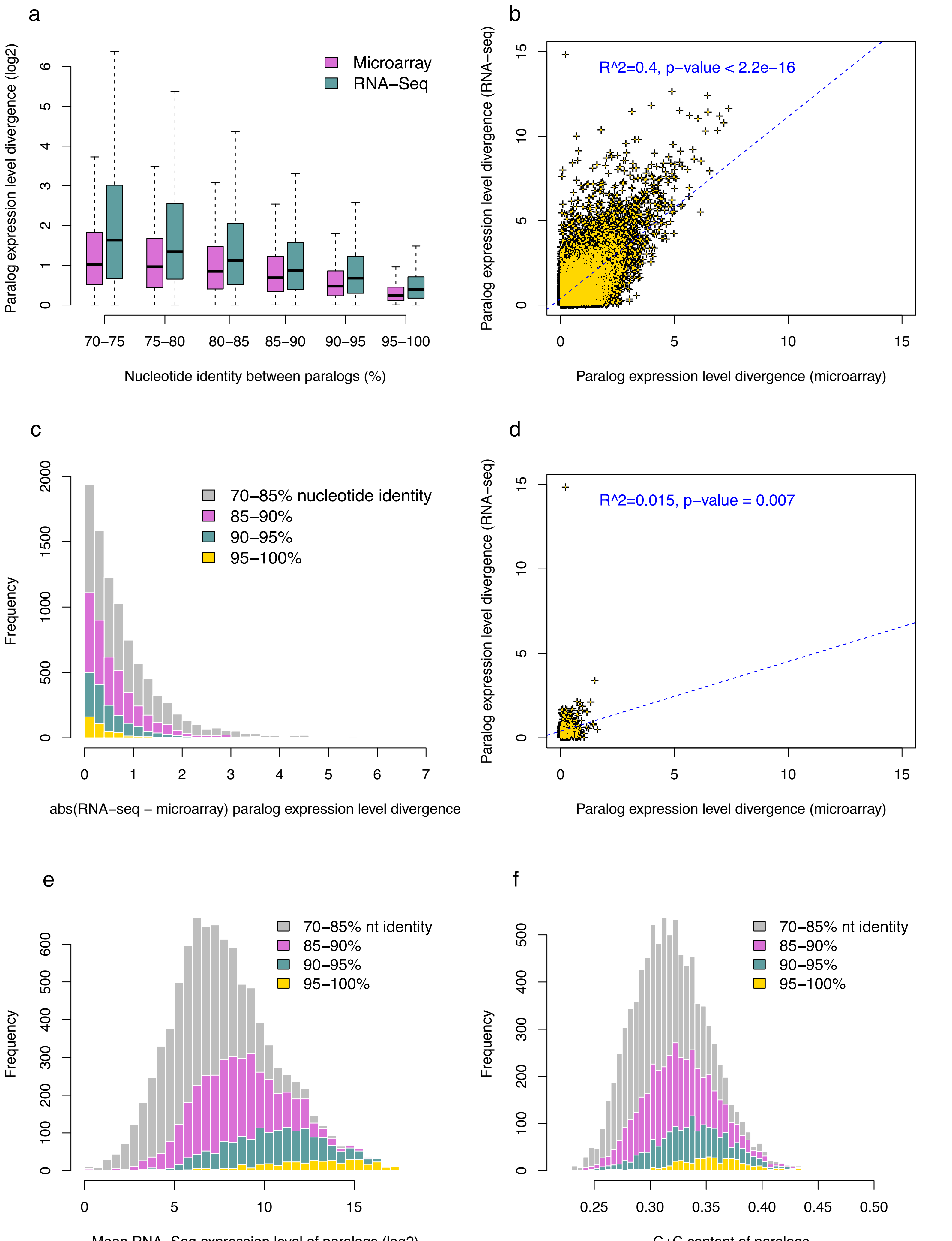

a
Paralog expression level divergence (log2)
Microarray
RNA−Seq
70−75
75−80
80−85
85−90
90−95
95−100
Nucleotide identity between paralogs (%)
b
Paralog expression level divergence (RNA−seq)
R^2=0.4, p−value < 2.2e−16
Paralog expression level divergence (microarray)
c
Frequency
70−85% nucleotide identity
85−90%
90−95%
95−100%
abs(RNA−seq − microarray) paralog expression level divergence
d
Paralog expression level divergence (RNA−seq)
R^2=0.015, p−value = 0.007
Paralog expression level divergence (microarray)
e
Frequency
70−85% nt identity
85−90%
90−95%
95−100%
Mean RNA−Seq expression level of paralogs (log2)
f
Frequency
70−85% nt identity
85−90%
90−95%
95−100%
G+C content of paralogs

Figure S7

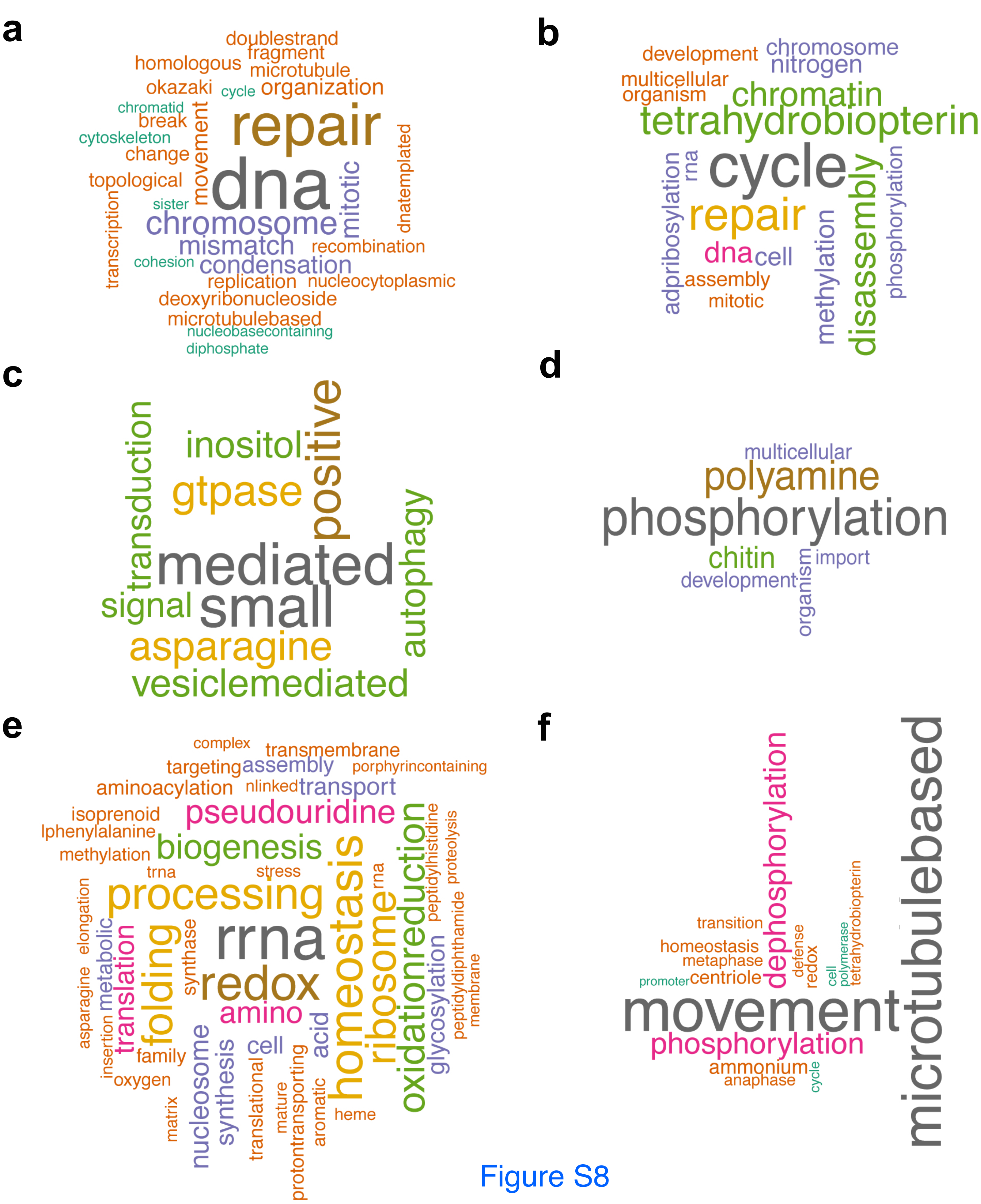
a
doublestrand
fragment
homologous
microtubule
okazaki
cycle
organization
chromatid
break
cytoskeleton
movement
repair
change
topological
dna
mitotic
dnatemplated
sister
transcription
chromosome
mismatch
recombination
cohesion
condensation
replication
nucleocytoplasmic
deoxyribonucleoside
microtubulebased
nucleobasecontaining
diphosphate
b
development
chromosome
nitrogen
multicellular
organism
chromatin
tetrahydrobiopterin
adpribosylation
rna
cycle
disassembly
phosphorylation
repair
dna
cell
methylation
assembly
mitotic
c
transduction
inositol
positive
gtpase
mediated
autophagy
signal
small
asparagine
vesiclemediated
d
multicellular
polyamine
phosphorylation
chitin
import
development
organism
e
complex
transmembrane
targeting
assembly
porphyrincontaining
aminoacylation
nlinked
transport
isoprenoid
pseudouridine
lphenylalanine
methylation
biogenesis
trna
stress
processing
homeostasis
rna
peptidylhistidine
proteolysis
elongation
oxidationreduction
ribosome
peptidyldiphthamide
asparagine
metabolic
translation
folding
synthase
rrna
redox
glycosylation
membrane
amino
acid
insertion
family
cell
oxygen
nucleosome
synthesis
translational
mature
protontransporting
aromatic
heme
matrix
f
dephosphorylation
microtubulebased
transition
homeostasis
metaphase
defense
redox
cell
polymerase
tetrahydrobiopterin
promoter
centriole
movement
phosphorylation
ammonium
anaphase
cycle

Figure S8
